# Assessing the Reliability and Credibility of Industry Science and Scientists

**DOI:** 10.1289/ehp.8417

**Published:** 2005-10-06

**Authors:** Craig S. Barrow, James W. Conrad

**Affiliations:** 1 Dow Chemical Company, Washington, DC, USA; 2 American Chemistry Council, Arlington, Virginia, USA

**Keywords:** bias, chemical industry, conflict of interest, industry science, industry scientists

## Abstract

The chemical industry extensively researches and tests its products to implement product stewardship commitments and to ensure compliance with governmental requirements. In this commentary we argue that a wide variety of mechanisms enable policymakers and the public to assure themselves that studies performed or funded by industry are identified as such, meet high scientific standards, and are not suppressed when their findings are adverse to industry’s interests. The more a given study follows these practices and standards, the more confidence one can place in it. No federal laws, rules, or policies express a presumption that scientific work should be ignored or given lesser weight because of the source of its funding. To the contrary, Congress has consistently mandated that agencies allow interested or affected parties to provide information to them and fairly consider that information. All participants in scientific review panels should disclose sources of potential biases and conflicts of interest. The former should be considered in seeking a balanced panel rather than being used as a basis for disqualification. Conflicts of interest generally do require disqualification, except where outweighed by the need for a person’s services. Within these constraints, chemical industry scientists can serve important and legitimate functions on scientific advisory panels and should not be unjustifiably prevented from contributing to their work.

The business of chemistry takes very seriously its responsibility to protect people and the environment throughout the entire life cycle of its products. This commitment is embodied in the product stewardship aspects of Responsible Care [American Chemistry Council (ACC) 2005a], the ACC’s initiative to continuously improve its members’ environmental, health, safety, and security performance, as well as the Long-Range Research Initiative (ACC 2005b), ACC’s voluntary initiative to fund research to increase understanding of the potential impacts chemicals may have on human health and the environment. Support for both programs is a condition of ACC membership. Also, a comprehensive set of U.S. and foreign government rules strictly regulate how chemicals are developed, manufactured, distributed, and used. The chemical industry conducts extensive research and testing on its products to implement product stewardship commitments and to ensure compliance with those governmental requirements. This work is conducted both directly by companies and indirectly through contracts with and grants to external scientists and research institutions. Consequently, the products of chemistry are among the most thoroughly evaluated and regulated in commerce. The research and testing conducted by the chemical industry are absolutely necessary, as they provide information on the potential health and environmental risks of substances that manufacturers, users, and government agencies all rely on to determine the conditions under which chemical products can be safely used.

In recent years some have questioned the reliability and credibility of public health and environmental research conducted or funded by the chemical industry, suggesting that industry research is fundamentally conflicted and hence unreliable (Devine 2001; Sass et al. 2005). These critics also challenge the legitimacy of allowing scientists employed or funded by industry to participate on scientific review panels (Center for Science in the Public Interest and Environmental Working Group 2004; Devine 2001; Greer and Steinzor 2002; Sass et al. 2005). Both contentions are mistaken and counterproductive to societal interests.

## Chemical Industry Science

Scientific studies conducted or funded by the chemical industry have long been acknowledged by government agencies, nongovernmental organizations, and the scientific community at large as necessary and valuable contributions to the understanding of potential public health and environmental effects related to the manufacture and use of chemicals [Environmental Defense 1998; Health and Environmental Sciences Institute 2005; U.S. Environmental Protection Agency (EPA) 2005d]. These same groups generally also recognize that, historically and for the foreseeable future, the costs associated with conducting chemical product testing have been and will be borne largely by industry, not the public sector, reflecting both the free enterprise view that those who benefit from an activity should bear the costs of that activity as well, and chronic resource limitations on the public sector.

A wide variety of mechanisms exists by which policymakers and the public they serve can assure themselves that studies performed by or funded by industry are identified as such, meet high scientific standards, and are not suppressed when their findings are adverse to industry’s interests. These practices and standards include the following:

- The ability of test sponsors to contractually authorize investigators—regardless of the results—to submit the investigators’ findings for publication in the peer-reviewed scientific literature without sponsor approval. This is the policy of ACC’s Long-Range Research Initiative.
- The practice of *Environmental Health Perspectives* and virtually all other scientific journals to require disclosure of funding sources.
- Peer review, which both government agencies and private entities may conduct or fund.
- The U.S. EPA requirement that all studies required to be submitted in connection with chemical regulation and pesticide statutes be conducted in accordance with U.S. EPA-approved guidelines for test protocols and Good Laboratory Practice (GLP) regulations (U.S. EPA 2005b, 2005c), which entail full availability to government authorities of the raw, quality-assured data files for review and audit. Research required to be submitted to regulatory agencies in member countries of the Organisation for Economic Cooperation and Development (OECD) must also follow OECD GLP principles (OECD 2005), which serve to substantiate the high quality and validity used for determining the safety of chemicals and chemical products in those countries.
- Information Quality Act (2000) guidelines issued by all federal agencies. These guidelines require scientific data to meet applicable standards for accuracy, reliability, and lack of bias and that apply to privately generated information when agencies rely on it for regulatory purposes (Office of Management and Budget 2002; U.S. EPA 2002a).
- U.S. EPA requirements under chemical and pesticide regulatory statutes (Federal Insecticide, Fungicide and Rodenticide Act 1972; Toxic Substances Control Act 1976), and similar global equivalents, that chemical manufacturers and pesticide registrants provide the U.S. EPA and its equivalents with timely notification of any adverse effects findings.
- The prospect of tort liability for suppression of adverse research findings.
- Finally, and most fundamentally, the scientific process itself through which different investigators attempt to reproduce the findings of others—a process that has led to the retraction of papers for which results could not be reproduced (McLachlan 1997).

The more a given study follows the above practices and standards, the more confidence one can place in it. In particular, the use of U.S. EPA-approved test protocols helps ensure that high standards of quality are being followed. Such protocols have been validated by the U.S. EPA and chosen after extensive and careful review to determine that the results would provide reproducible information suitable for regulatory decision making. Use of such protocols helps to produce a degree of certainty regarding the reliability and relevance of test results, which in turn provides the confidence necessary for making safety and regulatory determinations. Similarly, when research studies adhere to GLPs, as is the norm for industry health and environmental studies, reviewers and those acting on the science may have a high degree of confidence that the experimenters adhered to the specific and detailed experimental protocol employed, took all of the steps and measurements claimed to be taken during conduct of the study itself, and accurately reported the test results (Anderson et al. 2001).

No federal laws, rules, or policies express a presumption that scientific work should be ignored or given lesser weight because of the source of its funding. To the contrary, an entire body of federal law embodies a congressional mandate that agencies allow interested or affected parties to provide information to them and fairly consider that information. Primary among these is the Administrative Procedure Act (1946); others include the Information Quality Act (2000), the Federal Advisory Committee Act (1972), the Federal Register Act (1935), the Regulatory Flexibility Act (1980), and the Paperwork Reduction Act (1980).

Ironically, the proliferation of articles and presentations impugning the merits of industry science is having the effect of subjecting that science to much greater public and agency scrutiny than is applied to science conducted or funded by government or nonprofit entities (Miller 2005). Such scrutiny can only increase the likelihood that any flaws in the work will be identified.

Ultimately, all scientific research must stand or fall on its merits. Researchers should disclose their sources of funding because in cases where those sources have a potential interest in a question addressed by the research, others may want to scrutinize that research with heightened care to determine the extent to which it followed the practices and standards discussed above. However, it is unscientific (Society of Toxicology 1997), as well as unfair, to disregard or discount a study based solely on which investigator or institution conducted or funded it.

## Chemical Industry Scientists

The chemical industry’s commitment to scientific research and product testing includes engaging the highest quality scientists. Our scientists have national and international stature in the scientific community, as reflected by their inclusion on such authoritative bodies as the National Academies’ Board on Environmental Studies and Toxicology (National Academies 2005) and the U.S. EPA Science Advisory Board (U.S. EPA 2005a). These scientists have expert knowledge of the chemicals their employers manufacture and fully appreciate the value of their contributions—as objective, trained scientific experts—to the development and interpretation of the science needed to evaluate the health and environmental effects of their products. As members of professional associations such as the Society of Toxicology, industry scientists adhere to both personal and professional commitments to act in accordance with the codes of ethics of their professions (Society of Toxicology 1985).

As noted above, some have argued that scientists employed or funded partially by industry should not be permitted to serve on private or governmental review panels or similar bodies due to an asserted conflict of interest. Any discussion of this issue must carefully distinguish between conflict of interest and bias.

Federal rules issued under the Ethics in Government Act (Office of Government Ethics 1997) provide that true conflicts of interest are limited to instances where a person has a concrete financial interest in the subject being addressed. A conflict might occur, for example, in the case of an employee of a business that generates significant revenues from a product, if that employee is tapped to review a government assessment of that product. A conflict of interest could also occur in the case of a “public interest” representative, particularly if that representative is also serving as an expert witness in connection with ongoing litigation over the same subject matter. It is important to note that these government ethics rules still allow a person with a financial interest to serve where “the need for the individual’s services outweighs the potential for a conflict of interest.”

By contrast, bias (or “partiality,” under government ethics rules) is both unavoidable and unobjectionable. As the National Academies explain, bias derives from

points of view or positions that are largely intellectually motivated or that arise from the close identification or association of an individual with a point of view of a particular group. (National Academies 2001)

Similarly, a U.S. EPA Science Advisory Board committee has stated that

[a]lthough it is possible to avoid conflict of interest, avoidance of bias is probably not possible. All scientists carry bias due, for example, to discipline, affiliation and experience. (U.S. EPA 2000)

Fifteen past presidents of the Society of Toxicology have written in *Risk Policy Report* 2002 that

[o]f course, all scientists have biases; acknowledging this, we as a society must be aware of those biases and seek to ensure balance in the scientific panels whose task is to provide the best possible technical review of complex, important issues.

Finally, any evaluation of the role of industry scientists in governmental processes must consider the federal laws referenced above that empower interested persons to have input into those processes. Particularly relevant in this context is the Federal Advisory Committee Act (1972), which requires advisory committees to be “balanced” and thus should prohibit both exclusion of, as well as domination by, any interest (Office of Government Ethics 1997).

Expertise is the touchstone that guides the procedures followed by both the National Academies (2001) and the U.S. EPA Science Advisory Board (U.S. EPA 2002b). The National Academies’ current policy (National Academies 2003) is a particularly useful and appropriate statement of the relevant issues, for the following reasons:

- It emphasizes that knowledge, training, and experience are the foremost considerations and that no one should be appointed to a panel to represent a particular point of view or special interest.
- It clarifies that “[f]or some studies . . . it may be important to have an ‘industrial’ perspective or an ‘environmental’ perspective,” not because these “sides” need to be represented, but
  because such individuals, through their particular knowledge and experience, are often vital to achieving an informed, comprehensive, and authoritative understanding and analysis of the specific problems and potential solutions to be considered by the committee.
- It notes that “conflict of interest” ordinarily refers to “financial interests” and that these can arise from any quarter, including regulated entities, the government, and private organizations.
- It explains that biases should not be disqualifying—even when a person works for a company with “a general business interest in” the subject of the panel—unless the person
  is totally committed to a particular point of view and unwilling, or reasonably perceived to be unwilling, to consider other perspectives or relevant evidence to the contrary.

Such a case of bias would seem to be present in the report of Sass et al. (2005), for example, which argues that peer-review panels involving industry scientists are not “scientifically credible.” The article acknowledges funding from the Beldon Fund, which awarded the authors’ employer (the Natural Resources Defense Council) a 3-year, $210,000 grant

[t]o implement [Natural Resources Defense Council’s] Public Interest Service Initiative, a campaign to remove industry-funded scientists from EPA advisory boards and to appoint scientists dedicated to protecting human health and the environment. (Beldon Fund 2001)

The Office of Management and Budget’s recent peer-review guidelines agree with the National Academies and the U.S. EPA that

the most important factor in selecting reviewers is expertise: ensuring that the selected reviewer has the knowledge, experience and skills necessary to perform the review. (Office of Management and Budget 2005)

Consistent with these authoritative sources, scientists employed or funded by industry should be eligible to participate in peer-review panels and similar bodies just like any other scientists, based on the knowledge, training, and experience they bring to the body. All participants in such bodies should disclose sources of potential biases and conflicts. Potential biases should be considered in seeking a balanced panel rather than being used as a basis for disqualifying an expert. True conflicts of interest generally do require disqualification, except when outweighed by the need for a person’s services.

## Conclusion

The chemical industry takes seriously its central responsibility to conduct or fund research and testing of chemicals for use in the risk assessment processes. The scientific work it conducts and funds has an important and appropriate role in the development of health and environmental information. Its scientists can serve important and legitimate functions on scientific advisory panels. Frequently, they can provide unique knowledge and insight concerning the chemical in question and therefore should not be unjustifiably prevented from contributing to the work of such panels.
